# Astaxanthin improves fatty acid dysregulation in diabetes by controlling the AMPK-SIRT1 pathway

**DOI:** 10.17179/excli2023-6132

**Published:** 2023-06-14

**Authors:** Sana Taghiyar, Fatemeh Pourrajab, Mohammad Hosein Aarabi

**Affiliations:** 1 Department of Clinical Biochemistry, International Campus, Shahid Sadoughi University of Medical Science, Yazd, Iran; 2 Reproductive Immunology Research Center, Shahid Sadoughi University of Medical Sciences, Yazd, Iran; 3 Department of Clinical Biochemistry, School of Pharmacy and Pharmaceutical Sciences, Isfahan University of Medical Sciences, Isfahan, I.R. Iran

**Keywords:** astaxanthin, diabetes mellitus, SIRT1, AMPK, fatty acids profile

## Abstract

Due to the rising prevalence of metabolic disorders, including type 2 diabetes (T2DM), new prevention and treatment strategies are needed. The aim was to examine the effect of astaxanthin (AST) on the major regulatory metabolism pathway SIRT-MAPK and fatty acid (FA) profile of plasma in patients with T2DM. This clinical trial included 68 T2DM patients randomly assigned to receive 10 mg/day of oral AST (n = 34) or placebo (n = 33) for 12 weeks. The expression level of SIRT1, AMPK activity, and the level of fatty acids in the serum were examined. The results showed that AST could modify the serum levels of saturated fatty acids (SFA) and polyunsaturated fatty acids (PUFA), particularly that of Arachidonic acid, from 11.31±0.35 to 8.52±0.72 %. Also, AST increased the expression and activity levels of SIRT1 and AMPK, respectively. Pearson analysis also revealed a significant association between AMPK activity and Linoleic acid serum (LA) levels (~ -0.604, p~0.013). AST can modify the FA profile of plasma by inducing metabolizing cells to uptake them. Also, it can activate the SIRT-AMPK pathway related to metabolism regulation.

See also Figure 1(Fig. 1).

## Introduction

Metabolic syndrome and diabetes are an increasingly expanding global epidemic that poses a grave threat. Type 2 diabetes mellitus (T2DM) is a type of metabolic syndrome defined by elevated glucose and lipid levels in the blood, which is caused by multiple factors. AMPK plays a vital function in energy metabolism, including lipid, glucose, and protein metabolism, and is also essential for mitochondrial biogenesis and quality control. In recent years, AMPK has garnered great attention for its crucial function as a target of metformin and thiazolidinediones for treating T2DM and associated metabolic illnesses (Cantó et al., 2009[7]). Additionally, in the skeletal muscles of AST-treated obese and lean mice, the gene expression levels of Sirtuin, such as SIRT1, were elevated, which is strongly associated with AMPK activation (Brandauer et al., 2013[4]). According to cell culture and animal research, astaxanthin may affect FA metabolism in the muscle by modulating AMPK activation (Nishida et al., 2020[22]). Additional studies have also revealed a direct relationship between SIRT1 and fatty acid metabolism, indicating that therapeutic techniques to modify SIRT1 activity may effectively treat metabolic syndrome (Purushotham et al., 2009[25]). Moreover, AST possibly improves mitochondria function and induces an anti-oxidative effect through AMPK/Sirtuin/PGC-1α pathway (Nishida et al., 2022[21], 2020[22]). Alternatively, the expression of SIRT1 in T2DM patients may correlate with the glucose/lipid metabolism state (Song et al., 2011[28]). Current advances in the biological characteristics of antioxidants such as carotenoids suggest that these substances are not only capable of preventing diabetes and its complications but also of ameliorating them (Roohbakhsh et al., 2017[26]). AST, a xanthophyll carotenoid that stimulates endogenous antioxidant mechanisms by regulating gene expression, offers many advantages over other carotenoids (Ambati et al., 2014[1]; Murillo and Fernandez, 2016[20]). The anti-inflammatory properties of AST have also been approved (Fakhri et al., 2018[11]; Kim and Kim, 2019[16]; Yang et al., 2013[30]).

The effects of AMPK and SIRT1 on metabolism raise the issue of whether SIRT1 and AMPK activating medications effectively change the fatty acid profile of diabetes patients under pathological settings. Understanding the function of the SIRT1-AMPK pathway in diabetes and elucidating the regulatory impact of those on fatty acids, as well as the effect of the medicine on modifying the level of fatty acids through the SIRT1-AMPK route, is the primary objective of this study. Thus, we want to examine the effects of AST supplementation on the expression of SIRT1, AMPK activity, and fatty acid levels in individuals with type 2 diabetes and their connection.

## Materials and Methods

### Study population

Sixty-eight male and female patients with type II diabetes registered at the Isfahan Diabetes Research Center (associated with Isfahan University of Medical Sciences, Isfahan, Iran) were recruited in the study after completing a written consent form. A biostatistician established the size of the sample. Patients who visited the Diabetes Research Center at the Isfahan University of Medical Sciences met the inclusion criteria. Men and women aged 40 to 65 with type II diabetes who did not eat sources containing considerable levels of astaxanthin for 12 weeks were eligible for involvement in the trial. None of the participants had been given insulin. In this research, we adopted the set criteria by the American Diabetes Association standards (American Diabetes Association, 2020[2]). All participants were permitted to continue taking their oral diabetes medications (as prescribed by their doctor) during the trial, but they were not allowed to change their normal treatment regimen. Patients with the following conditions were excluded from this study: pregnancy or lactation; specific diseases and malignancies; kidney failure; heart disease; a thyroid disorder; and other inflammatory diseases; patients did not report taking any supplements within the six months prior to recruitment. Individuals who were taking supplements or medications for other conditions were eliminated. Before undergoing any study-related procedures, each patient completed an informed consent form. Any change in a participant's regular medication or sensitivity to the extract would result in removal from the trial.

### Study design

This research was intended as a random, double-blind, placebo-controlled, investigator-initiated experiment with allocation concealment. 10 mg of AST supplement from *H. pluvialis* was acquired from Waka Tani Health Nutrition Corporation in the United States (microalgae). Barij Essence Pharmaceutical Co., Iran, manufactured the placebo capsules. The form and color of the placebo capsules were similar to those of the AST capsules. The inactive substances in the supplement and placebo were dicalcium phosphate, microcrystalline cellulose, stearic acid, silicon dioxide, and magnesium stearate. Randomly, the 68 individuals were allocated into two groups. The placebo group (n = 33) got one 10 mg pill daily (containing the supplement's basic material, except AST). Group AST (n = 34) got one capsule daily containing 10 mg of AST for twelve weeks (based on previous studies and at the discretion of the endocrinologist). 

In order to maximize absorption, the subjects were asked to consume one capsule every day for 12 weeks after lunch without changing how they lived. Throughout the study, one of the researchers contacted each participant once a week to confirm compliance and handle any possible issues. After 12 weeks of astaxanthin treatment in diabetic individuals, alterations to the sirtuin1 gene, AMPK activity, and fatty acid profiles were evaluated. The Ethics Committee at Shahid Sadoughi University of Medical Sciences approved the study protocol with Ethics Code IR.SSU.MEDICINE.REC.1400.307, and was conducted by the Declaration of Helsinki. 

### Randomization and allocation concealment

Afterward, participants were assigned to one of two groups using a predetermined randomization procedure. An assistant used the block randomization approach to do the randomization, and stratified randomization was used to match individuals based on gender distribution. As a result, an equal number of subjects were randomly randomized to either AST or a placebo. The astaxanthin and placebo groups were provided in similar packets, allowing the principle investigator, sub-investigators, clinical trial staff, and research volunteers to be blinded.

### Anthropometric measurements

The patients were weighed while wearing minimum clothing and without shoes, and their standing height was assessed. The formula for calculating body mass index (BMI) is weight in kilograms divided by height in meters squared. To eliminate subjectivity, all measurements were taken by the same individual.

### Biochemical analysis

#### Blood sampling

The participants' venous blood was drawn following a 12-hour overnight fast at the beginning of the research, before the intervention, and again after the experiment, 12 weeks later. A fraction of the blood was utilized to evaluate the expression of the SIRT1 gene. Serum was isolated from the other half and utilized to assess AMPK activation and the profile of fatty acids.

#### Total RNA extraction for SIRT1

Mononuclear cells were extracted from peripheral blood by density gradient centrifugation using Ficoll-Paque. Genomic RNA was isolated from peripheral blood mononuclear cells (PBMC) using the RNX-Plus reagent (Cinnagen), followed by DNase I digestion (Takara Bio Inc), according to the user handbook. RNA quality was assessed using the Epoch™ Multi-Volume multi-sample spectrophotometer instrument reading at 230, 260 and 280 nm wavelengths.

#### cDNA synthesis and quantitative real-time PCR for SIRT1

For reverse transcription of mRNA to cDNA, an ANACELL (IranN) cDNA synthesis kit was employed. SIRT1 expression was measured using the SYBR Green PCR Kit (ANACELL, Iran). The expression data were normalized to the geometric mean expression level of the housekeeping gene GAPDH and calculated using the 2^−Δct^ and 2^−ΔΔct^ methods. Each sample was performed in triplicate, and each experiment was repeated thrice. The set of primers for particular genes is presented in Table 1(Tab. 1).

#### Measurements of AMPK activity

Using the Biorex (Iran) AMPK kit and a colorimetric method, the serum AMPK activity was determined.

#### Fatty acids measurement of serum

##### Lipid extraction

Serum lipids were isolated using the Folch technique (Folch et al., 1957[12]). 100 µL of serum samples were added to 3 mL of a 2:1 mixture of chloroform and methanol in a test tube. The tubes were vortexed after adding 100 µL BHT (5 mg/mL in chloroform) as an antioxidant and 100 µL heptadecanoic acid (1000 µg/mL in chloroform) as an internal standard (to measure the recovery rate). After adding 600 µL of 0.9 % NaCl solution, the tubes were mixed again. The phases were separated by centrifuging tubes at 1,000 rpm for three minutes. The lipid-containing lower chloroform phase was collected and transferred to another test tube. Anhydrous sodium sulfate was added to remove residual water, and the lipid extract was transferred to a 15-mL Teflon-lined screw cap tube and dried at room temperature using nitrogen.

##### Fatty acid methyl ester preparation

BF3 was used to manufacture fatty acid methyl esters according to the technique of Morrison and Smith (1964[19]). In a 15 mL Teflon-lined screw-cap tube, 1 mL hexane and 1 mL BF3/methanol (13 %) were added to the dried lipid extract and mixed well. The mixture was cooked in an oven at 100 °C for 45 minutes. Once the mixture had cooled, 0.5 mL of hexane and 0.5 mL of HPLC-grade water were added, shaken, and centrifuged. The percentage of methyl esters in the top hexane layer was then extracted using a Pasteur pipette and dried under nitrogen gas. Before GC analysis, fatty acid methyl esters were dissolved in 200 L hexane.

##### Calibration standards and chemical compounds

A commercially accessible standard combination of 37 fatty acid methyl esters (FAME) and a number of individual fatty acid methyl ester standards, including myristic acid (C14:0), palmitic acid (C16:0), stearic acid (C18:0), arachidic acid (C20:0), myristoleic acid (C14:1), palmitoleic acid (C16:1), oleic acid (C18:1n9), linoleic acid (C18:2n6), α-linolenic acid (ALA, C18:3n3), di-homo gamma-linolenic acid (DGLA, C20:3n6), Cis-11,14,17-eicosatrienoic acid (ETE, C20:3n3), arachidonic acid (AA, C20:4n6), cis-5,8,11,14,17- eicosapentaenoic acid (EPA, C20:5n3), Cis-4,7,10,13,16,19- docosahexaenoic acid (DHA, C22:6n3), and non-methylated heptadecanoic acid (HDA, C17:0) (as internal standard) (all obtained from Sigma Chemical Co., St. Louis, MO, USA) were used for calibration. Boron trifluoride methanol (BF3-M) and butylated hydroxytoluene (BHT) were purchased from Sigma Chemical Co. (St. Louis, MO). In contrast, all other reagents, chemical compounds, and solvents were purchased from Merck (Merck Co., Darmstadt, Germany).

##### Instrumentation, chromatography, and detection method

The fatty acid composition of serum was evaluated using gas chromatography. GC studies used the FID detector-equipped Younglin GC-FID system model Acme 6000 M (Young Lin Co., Hogye, Anyang, Korea). The injector and detector temperatures were maintained at 260 °C, and the flame was maintained using 40 milliliters per minute of hydrogen and 300 milliliters per minute of air. At a 28 mL/min flow rate, helium was used as the detector's auxiliary gas. Using an SP-2560 fused silica capillary column with a 100 m 0.25 mm 0.2 µm film thickness, chromatography was performed (Supelco Co., Bellefonte, PA). A steady flow of 18 cm/s of helium was employed as the carrier gas. For the analysis, 1 µL sample injections were conducted with a split ratio of 17:1. The oven temperature was designed to increase from 140 to 245 °C at a rate of 4 °C per minute after an initial time hold of 5 minutes and a final time hold of 15 minutes (the total analysis time was 45 min).

### Statistical analysis

One-way analysis of variance was used to compare groups at baseline and after treatment, while a student-paired t-test was employed to identify statistically significant changes in metabolic status and gene expression between pre- and post-treatment values. Using the Kolmogorov-Smirnov test, we checked whether the data followed a normal distribution. Pearson correlation was used to investigate inter-variable relationships. P < 0.05 was considered statistically significant for all experiments. SPSS was used for all statistical testing (Version 20, SPSS Inc).

## Results

### Participant characteristics

**Figure 1:**
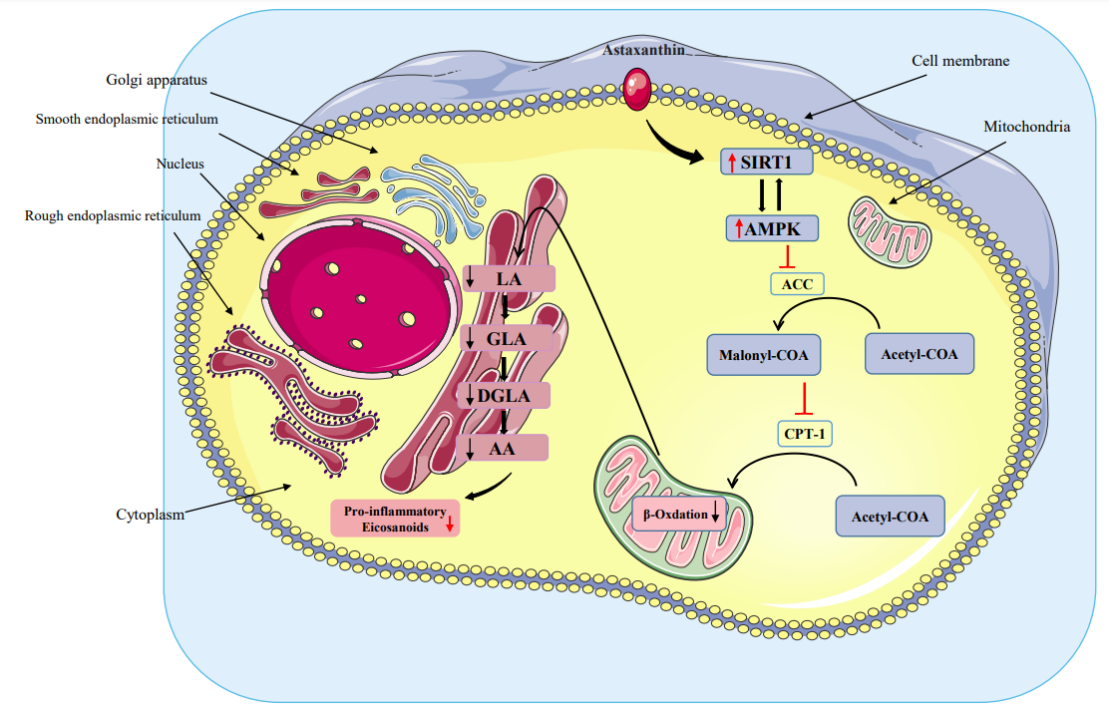
Graphical abstract The impact of astaxanthin on the fatty acid regulation via the AMPK-SIRT1 pathway. LA: linoleic acid, GLA: gamma-linolenic, DGLA: dihomo-gamma-linolenic acid, AA: arachidonic acid

Samples of 68 patients who participated and completed the study were examined and analyzed (see supplementary information Figure 1). There were no significant variations in age, gender, weight, or BMI between the two groups (see supplementary information Table 1). Participants should comply with the protocol instructions: before, after, and throughout the intervention, the dietary factors, including the total dietary energy, fat, protein, carbohydrate, and micronutrient consumption, should not be changed. Also, none of which are the finest natural sources of AST, including algae, yeast, wild sockeye salmon, trout, krill, and shrimp, should be consumed by our subjects. Thus, dietary sources of AST were restricted, and consumption of other carotenoids did not vary across research groups.

### AST induces the expression of the SIRT1 gene

**Figure 2:**
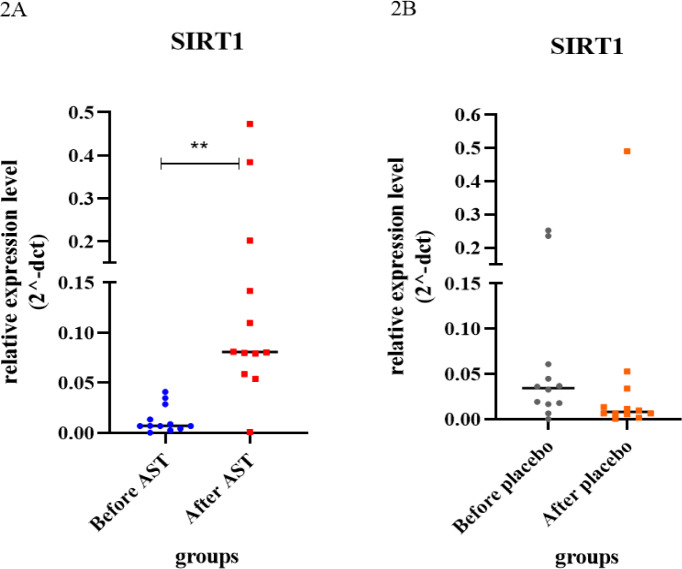
Analysis of the relative gene expression of SIRT1 in the placebo and astaxanthin groups, before and after the intervention, compared to the control gene GAPDH. A) The differences in mean values between the treatment groups were statistically significant; there was a difference between before and after intervention (p-value ~ 0.0022). B) The differences in mean values between the placebo groups were insufficient (p-value ~ 0.660).

According to the instruction, a significant increase of SIRT1 gene expression in PBMCs of blood was found in patients consuming AST. As measured by the 2^−ΔΔCT^ formula, the expression level of SIRT1 was considerably increased (fold change expression ~ 10.691; p-value ~ 0.0022) (Figure 2(Fig. 2), supplementary information Table 2). Also, the relative expression of SIRT1 was considerably higher in the AST-treated group compared to the other groups. (p-value ~ 0.0348) (Figure 4A).

### AST acts as AMPK activator

**Figure 3:**
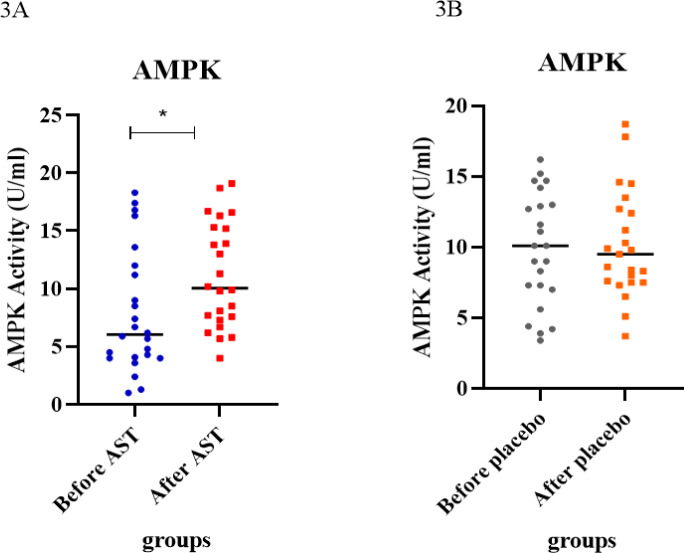
Investigation of AMPK activity in AST and placebo groups before and after supplementation. A) The differences in mean values between the treatment groups were statistically significant; there was a difference between before and after intervention (p-value ~ 0.02). B) The differences in mean values between the placebo groups were insufficient (p-value ~ 0.82).

**Figure 4:**
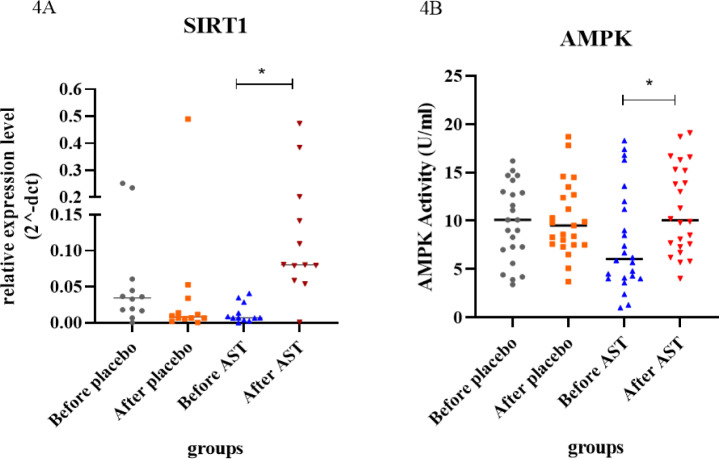
One-way ANOVA was used to examine the relative expression level of the SIRT1 gene & AMPK activity in the research groups. The activity of the AMPK was higher in the AST-treated group compared to the other groups. (P ~ 0.0586). The relative expression of SIRT1 was higher in the AST-treated group compared to the other groups. (p-value ~ 0.0348). The asterisk (*) indicates a substantial difference compared between the groups.

In this work, AST showed a significant increase in AMPK activity in diabetic subjects treated with astaxanthin. The level of AMPK enzyme in biochemical tests at the beginning and end of the study in the control group (placebo recipient) was 9.82 ± 1.42 and 10.15 ± 1.87, respectively. Also, the level of this enzyme in the astaxanthin group at the beginning and end of the study was 7.87 ± 1.14 and 11.14 ± 1.09, respectively. This shows no significant effect of the placebo on the activity level of this enzyme (P<0.82). In contrast, the findings showed that the use of AST supplements in the treatment of patients with type 2 diabetes, with a significant increase in the level of this enzyme after the intervention, was associated with the baseline values in these patients (P<0.02) (Figure 3A, 3B(Fig. 3)). Analysis of the data revealed that the activity of the AMPK was considerably higher in the AST-treated group compared to the other groups (P=0.0586) (Figure 4B(Fig. 4)).

### AST changes the serum profile of fatty acids in T2DM patients

Tables 2(Tab. 2) and 3(Tab. 3) depict the FAs profile of serum in participants. The content of individual FAs in serum was expressed as percentages of the total FAs identified. Most FAs detected in serum, save AA, did not vary substantially between the two groups. AA level, during the two weeks of therapy with a 10 mg dosage of astaxanthin, revealed a 24 % drop in average levels (from 11.31±0.35 to 8.52±0.72 %), which were statistically highly significant (p-value < 0.01) (Table 2(Tab. 2)). SFAs level after the two weeks of therapy with a 10 mg dosage of astaxanthin revealed a 1.27 % decrease minor in levels (from 33.02±1.68 to 32.60±1.16 %), which was a significant reduction in group AST compared to that in group placebo (p-value < 0.01) and also polyunsaturated fatty acids (PUFA) level after the two weeks of therapy, revealed an 11.65 % slight drop in levels (from 46.87±3.19 to 41.41±4.20 %) which was a significant reduction in group AST compared to that in group placebo (p-value < 0.01). There were no significant changes in monounsaturated fatty acids (MUFA), n-3 and n-6 fatty acids, and the ratio of fatty acids between the two groups (Table 3(Tab. 3)).

### Associations between FA in the serum of patients treated with AST

**Figure 5:**
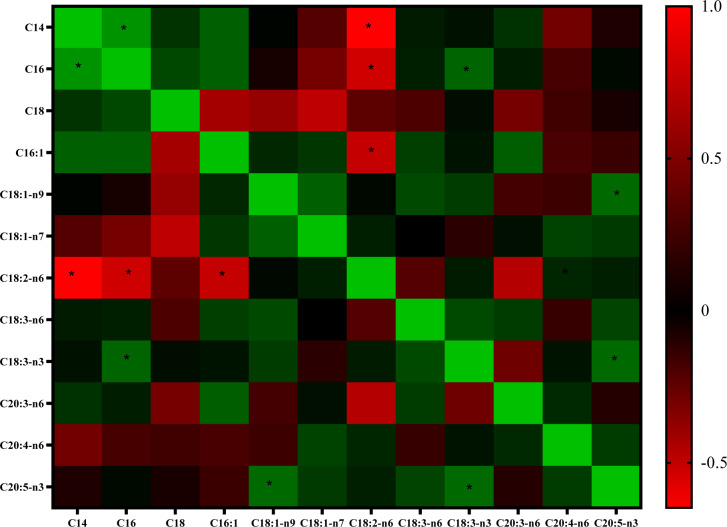
The color shift in the heat map means that red shows the indirect association, and green indicates the direct link between fatty acids. *Significant at *P* < 0.05.

LA levels were negatively connected (r =-0.604, P~0.013) with AMPK in the research population (n=67). Figure 5(Fig. 5) depicts a serum fatty acid association heat map (Figure 5(Fig. 5)).

See also the Supplementary data.

## Discussion

In the current investigation, we explored whether astaxanthin supplementation affected the AMPK-SIRT1 pathway in altering the plasma profile of fatty acids in type 2 diabetes patients. Our findings show that this supplement may impact the fatty acid profile in diabetes individuals via this mechanism. In the investigation of fatty acid profiles, arachidonic acid reduced dramatically following treatment with astaxanthin supplementation; on the other hand, a correlation was detected between SIRT1 and arachidonic acid levels.

SIRT1 is an essential regulator of lipid homeostasis, in particular fatty acid oxidation. SIRT1 is downregulated in various cells and tissues in insulin-resistant or glucose-intolerant conditions, according to recent research (de Kreutzenberg et al., 2010[10]). Additionally, it has been found that diminished SIRT1 activity causes diabetes and metabolic syndrome (Chalkiadaki and Guarente, 2012[9]). An important result in the current research is that the impact of SIRT1 on fatty acid metabolism is related to changed arachidonic acid. The AMPK-SIRT1 circuits interact dynamically. AMPK has been demonstrated to activate SIRT1, most likely via an increase in cellular NAD+ levels (Cantó et al., 2009[8]), whereas SIRT1 deacetylates the AMPK kinase LKB1, increasing AMPK phosphorylation and activation (Ivanov et al., 2008[14]).

Also, AMPK is a critical sensor of cellular energy status found in practically all eukaryotes. It is a heterotrimer with a catalytic α subunit and regulatory β and γ subunits (Hardie et al., 2016[13]). As a primary cellular energy sensor, AMPK activation generates various positive effects on glucose and lipid metabolism in peripheral tissues, such as skeletal muscle, liver, and adipose tissue (Zeng et al., 2015[31]). As demonstrated in our data, AST has enhanced AMPK activity. AMPK modulates the fatty acid metabolic pathway in multiple ways. As part of this regulatory pathway, AMPK targets acetyl-CoA carboxylase activity (ACC) and phosphorylates it, thereby inhibiting ACC activity (Park et al., 2002[23]). Since ACC catalyzes the carboxylation of acetyl-CoA to produce malonyl-CoA, a substrate for fatty acid biosynthesis, inhibition of ACC activity reduces fatty acid biosynthesis. The ability to transport the long-chain fatty acyl-CoA from the cytosol into the mitochondria, where it is oxidized to generate acetyl-CoA, is crucial to the regulation of fatty acid oxidation. This process' rate-limiting enzyme is carnitine palmitoyl transferase (CPT-1). CPT-1 catalyzes the transfer of the acyl group from acyl-CoA to carnitine, thereby preparing carnitine for transport from the cytosol into the mitochondria. Malonyl-CoA inhibits CPT-1 activity allosterically, thereby impeding the β-oxidation of fatty acids (McGarry and Brown, 1997[18]). In conclusion, ACC inhibition by AMPK reduces malonyl-CoA levels, facilitating fatty acid transport into mitochondria and increasing β-oxidation rates. Accordingly, astaxanthin supplementation may reduce arachidonic acid precursors by increasing the rate of β-oxidation. Subsequently, the level of arachidonic acid also decreased due to the reduction of its precursors through the reduction of beta-oxidation of fatty acids through the AMPK-SIRT1 pathway.

The n-6 fatty acid arachidonic acid (AA; 20:4n-6) gives rise to eicosanoid mediators that have established inflammation functions. AA metabolism is a long-recognized target for routinely used anti-inflammatory treatments (Calder, 2009[6]). AA and its lipid metabolites, eicosanoids, play a key role in inflammation-induced cell dysfunction and insulin resistance during diabetes (Luo and Wang, 2011[17]). Arachidonic acid may operate as a powerful negative regulator of glucose absorption (Tebbey et al., 1994[29]). Earlier research has indicated greater blood levels of arachidonic acid in diabetes participants than in normal matched controls (Simopoulos, 1997[27]). Fatty acids may play harmful implications for metabolic health. High fatty acid levels are always connected with obesity and type 2 diabetes (Bi et al., 2019[3]). The probable involvement of fatty acids in obesity and insulin resistance has been explored over several decades with disputed results. High quantities of fatty acids may prevent insulin from acting as an anti-lipolytic, causing insulin resistance and further increasing the release of fatty acids into the bloodstream (Jensen et al., 1989[15]). There are several different ways that fatty acids might affect inflammation. The arachidonic acid pathway is a critical regulator of the inflammatory response, and also, Eicosanoids generated from arachidonic acid have functions in inflammation (Calder, 2011[5]). Because of its molecular structure and arrangement in the plasma membrane, astaxanthin, a carotenoid of the xanthophyll group, has powerful antioxidant effects. This carotenoid also possesses significant anti-inflammatory action, which may be connected to its antioxidant function, and is implicated in the control of lipid and glucose metabolism (Pereira et al., 2021[24]).

Based on our research data, AST administration lowered the level of AA, which plays a vital role in inflammation. This impact may be via the AMPK-SIRT pathway, which is one of the efficient mechanisms in regulating fatty acid metabolism (Figure 1(Fig. 1), graphical abstract). The strengths of the current research include the GC-FID analysis of FAs levels and the investigation of possible confounding effects of numerous variables, including clinical risk factors and medication usage. Regrettably, our findings should be taken with various caveats in mind. For starters, we did not gather the full dietary data needed to estimate FA consumption in patients. Nonetheless, given the considerable interpatient variation in metabolism and absorption, circulating levels of FAs may be more essential to health than food intake. Second, since all research patients were recruited from clinics in Isfahan in Iran, the link between FAs levels and variables may not generalize to other nations, patient groups, or the general public. To our knowledge, this is the first research that analyzes AST effects on fatty acid profile, its link with the AMPK-SIRT pathway in T2DM, and the potential benefit of AST supplementation on diabetic patients. Additionally, future big sample-size investigations are needed to get superior results, and our findings should be regarded as preliminary, and the demand for further studies is advised.

## Conclusion

In this work, we studied whether AST may suppress fatty acid-induced inflammation by explaining the mechanism and the AMPK-SIRT pathway. However, further larger sample-size research is necessary to validate these results.

## Declaration

### Ethics approval

This research is part of a registered protocol in the Iranian Registry of Clinical Trials (IR.SSU.MEDICINE.REC.1400.307), authorized by the Yazd University of Medical Sciences Research Ethics Committee.

### Acknowledgment

The present research is extracted from the Ph.D. thesis of Ms. Sana Taghiyar.

### Conflict of interest

The authors state that there is no conflict of interest.

### Funding

This research received no specific grant from funding agencies in the public, commercial, or not-for-profit sectors.

### Declaration of competing interest

- Ethical Approval and Consent to participate (NA)

- Consent for publication (NA)

- Availability of supporting data (Yes)

- Competing interests (NO)

- Funding (NO)

### Authorship contributions

MHA and ST designed the experiments; ST performed experiments and collected data; ST, MHA and FP discussed the results and strategy; MHA and FP supervised, directed and managed the study; ST, MHA and FP finally approved the version to be published.

## Supplementary Material

Supplementary information

Supplementary data

## Figures and Tables

**Table 1:**
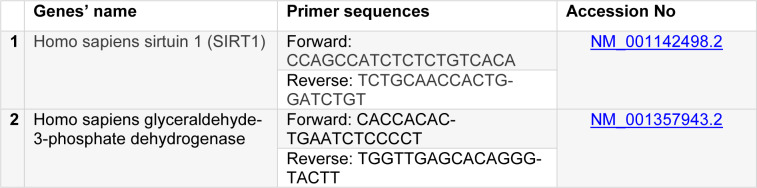
List of primer sets used in real-time polymerase chain reaction assays for SIRT1 and the housekeeping gene, GAPDH

**Table 2:**
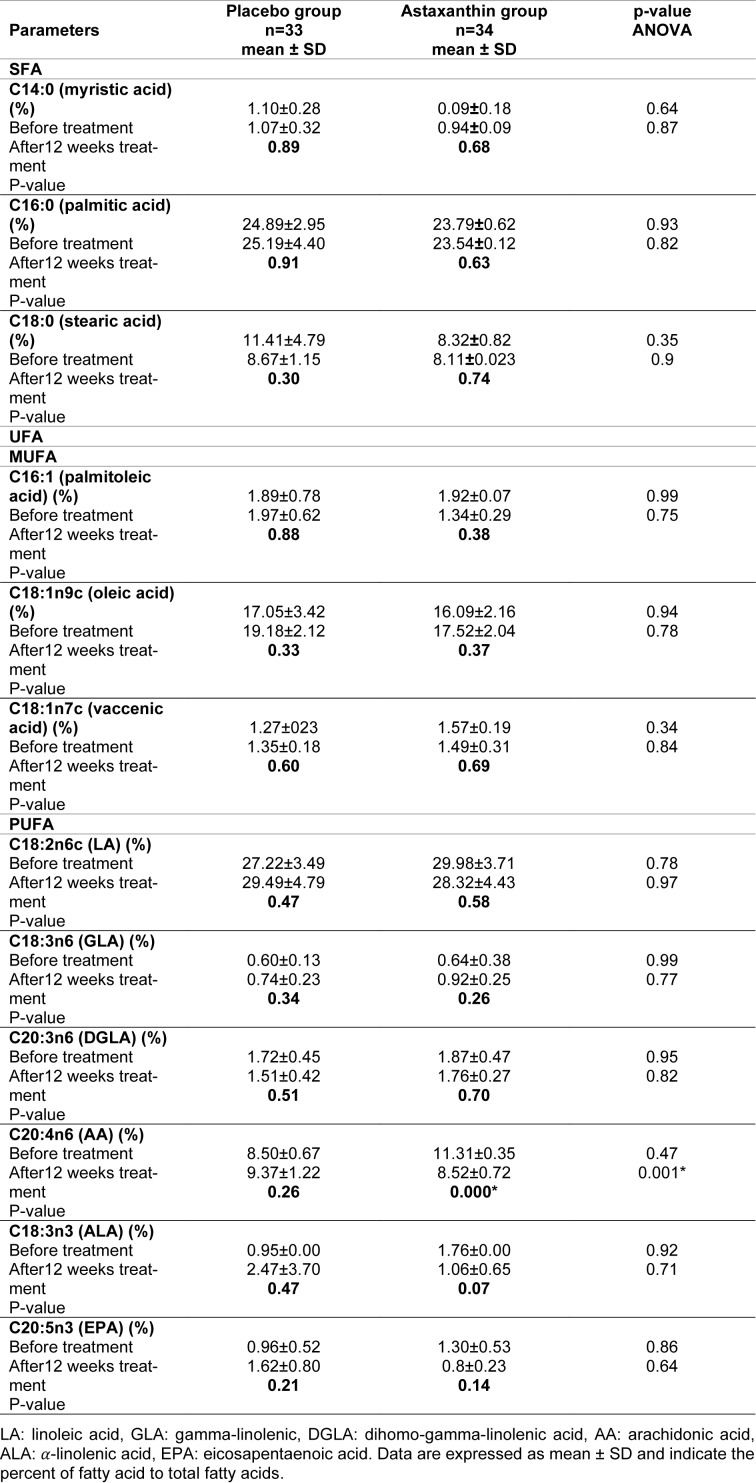
Profiles of fatty acids in the serum of patients with type 2 diabetes

**Table 3:**
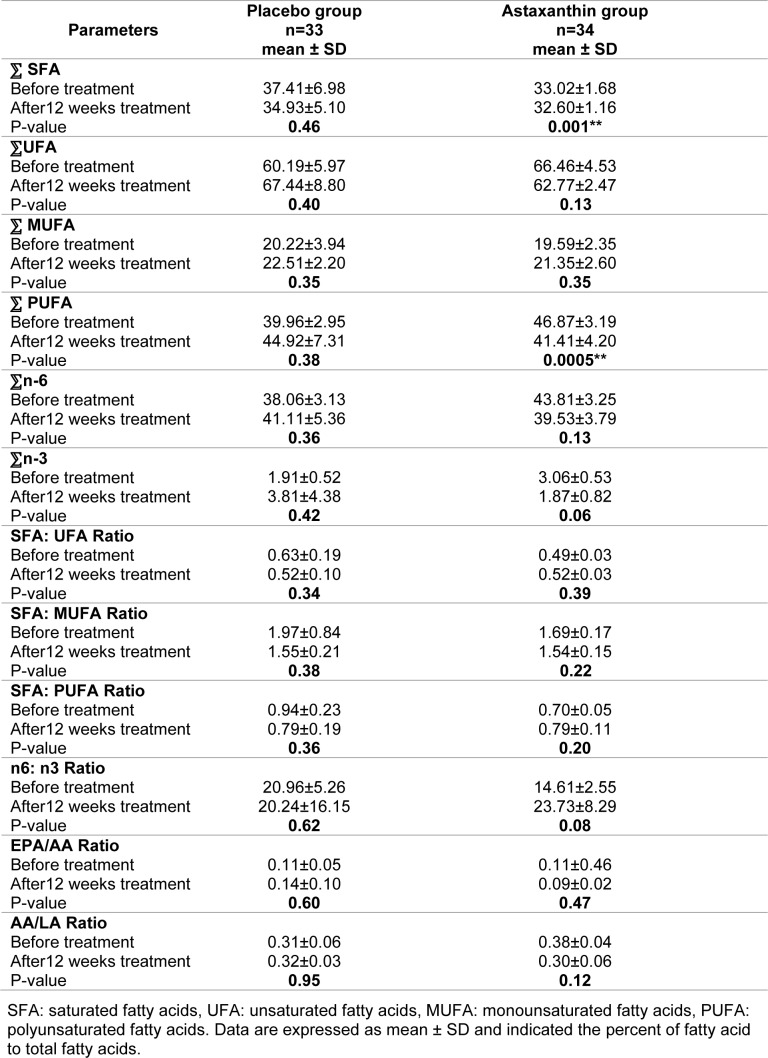
Distribution of total saturated fatty acid, total unsaturated fatty acid, and ratios in serum of type 2 diabetes patients between placebo (n=33) and astaxanthin group (n=34)
