# Supplementary material for: Astaxanthin improves fatty acid dysregulation in diabetes by controlling the AMPK-SIRT1 pathway
Source: EXCLI J. 2023 Jun 14;22:502–15. doi: 10.17179/excli2023-6132 (PMC10391612; doi:10.17179/excli2023-6132)
Supplement: Supplementary information [file EXCLI-22-502-s-001.pdf]

*EXCLI Journal 2023;22:502-515 – ISSN 1611-2156*
*Received: April 25, 2023, accepted: June 06, 2023, published: June 14, 2023*

## Supplementary information to:

## Original article:

# ASTAXANTHIN IMPROVES FATTY ACID DYSREGULATION IN DIABETES BY CONTROLLING THE AMPK-SIRT1 PATHWAY

Sana Taghiyar[1], Fatemeh Pourrajab[2*] Mohammad Hosein Aarabi[3]

[1] Department of Clinical Biochemistry, International Campus, Shahid Sadoughi University of Medical Science, Yazd, Iran
[2] Reproductive Immunology Research Center, Shahid Sadoughi University of Medical Sciences, Yazd, Iran
[3] Department of Clinical Biochemistry, School of Pharmacy and Pharmaceutical Sciences, Isfahan University of Medical Sciences, Isfahan, I.R. Iran

* **Corresponding author:** Fatemeh Pourrajab, Reproductive Immunology Research Center, Shahid Sadoughi University of Medical Sciences, Yazd, Iran.
E-mail: mina_poorrajab@yahoo.com

https://dx.doi.org/10.17179/excli2023-6132

This is an Open Access article distributed under the terms of the Creative Commons Attribution License (http://creativecommons.org/licenses/by/4.0/).

*EXCLI Journal 2023;22:502-515 – ISSN 1611-2156*
*Received: April 25, 2023, accepted: June 06, 2023, published: June 14, 2023*

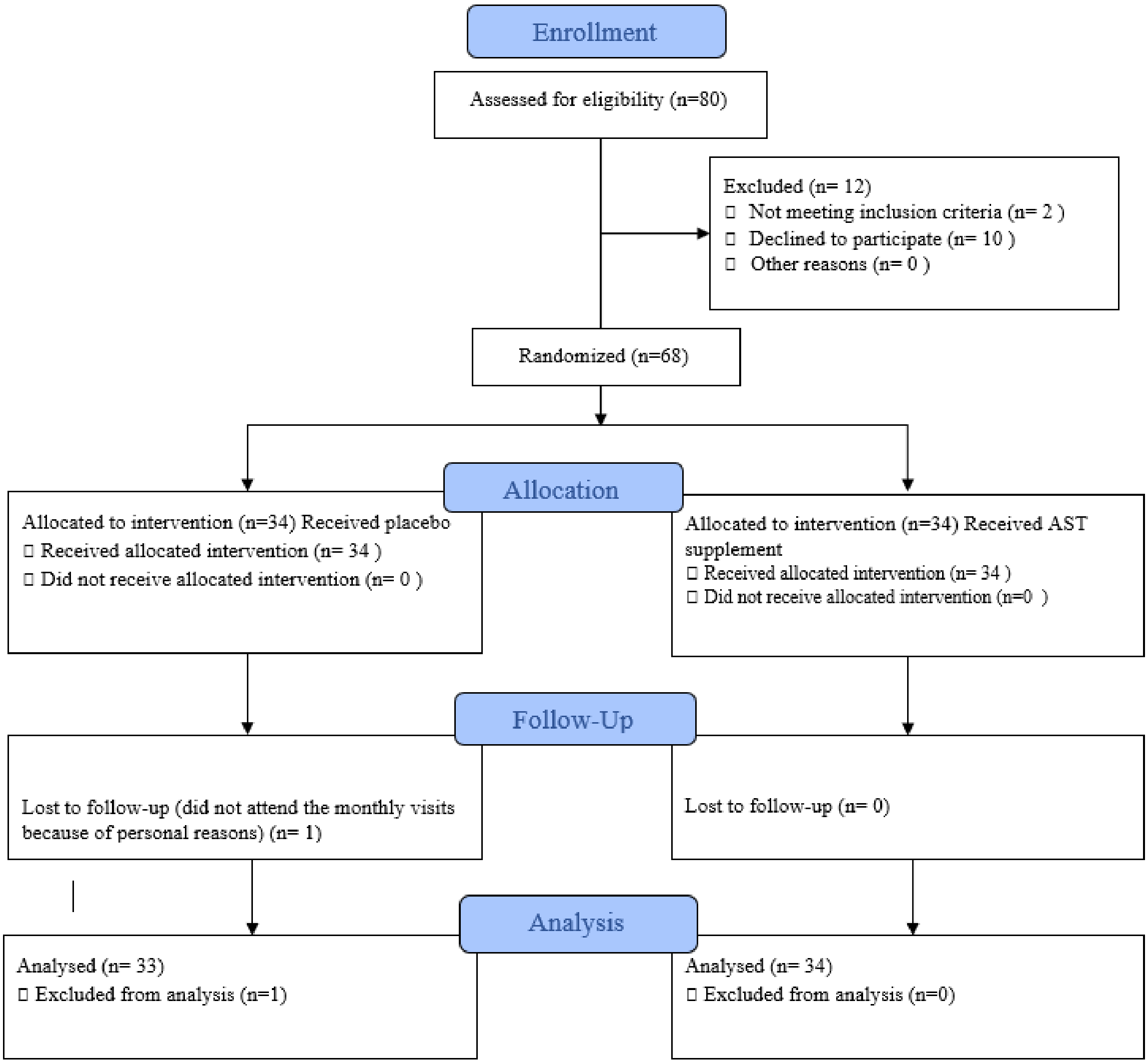


**Supplementary Figure 1:** Diagram depicting all people evaluated for eligibility, participated in the astaxanthin trial, randomized to various treatments, and included in analysis

EXCLI Journal 2023;22:502-515 – ISSN 1611-2156
Received: April 25, 2023, accepted: June 06, 2023, published: June 14, 2023

**Supplementary Table 1:** The anthropometric statistics of the type 2 diabetes study population

| Parameters | Placebo group n=33 | Astaxanthin group n=34 | P-value |
|---|---|---|---|
| **Age (years)** | 50.48 ± 7.35 | 52.88 ± 7.23 | 0.266 |
| **Sex (M/F)** | 13 (56.5%) | 9 (37.5%) | 0.191 |
| **Body mass index** | | | 0.1638 |
| Before treatment | 27.70 ± 3.65 | 29.64 ± 4.28 | |
| After12 weeks treatment | 27.73 ± 3.55 | 29.54 ± 4.30 | |
| P-value | 0.9802 | 0.9334 | |

Values are means ± SD of patients. *P < 0.05 for within- and between-group change from baseline to 12 weeks. Paired t-tests were used to compare values before and after treatment in each group, whereas differences between the two groups were analyzed by one-way ANOVA. Any P-value < 0.05 was considered statistically significant.

**Supplementary Table 2:** Analysis of relative gene expression of SIRT1 in the placebo and astaxanthin groups before and after intervention

| | Placebo group (n = 33) | Astaxanthin group (n = 34) | P-value |
|---|---|---|---|
| **Fold change (FC) of SIRT1 ($2^{-\Delta\Delta CT}$)** | 0.9797 | 10/6918 | 0/034* |

Note: * P-value < 0.05
