# Supplementary material for: Astaxanthin improves fatty acid dysregulation in diabetes by controlling the AMPK-SIRT1 pathway
Source: EXCLI J. 2023 Jun 14;22:502–15. doi: 10.17179/excli2023-6132 (PMC10391612; doi:10.17179/excli2023-6132)
Supplement: Supplementary data [file EXCLI-22-502-s-002.pdf]

EXCLI Journal 2023;22:502-515 – ISSN 1611-2156
Received: April 25, 2023, accepted: June 06, 2023, published: June 14, 2023

Supplementary data to:

Original article:

ASTAXANTHIN IMPROVES FATTY ACID DYSREGULATION IN DIABETES BY CONTROLLING THE AMPK-SIRT1 PATHWAY

Sana Taghiyar[1], Fatemeh Pourrajab[2*] Mohammad Hosein Aarabi[3]

[1] Department of Clinical Biochemistry, International Campus, Shahid Sadoughi University of Medical Science, Yazd, Iran
[2] Reproductive Immunology Research Center, Shahid Sadoughi University of Medical Sciences, Yazd, Iran
[3] Department of Clinical Biochemistry, School of Pharmacy and Pharmaceutical Sciences, Isfahan University of Medical Sciences, Isfahan, I.R. Iran

* **Corresponding author:** Fatemeh Pourrajab, Reproductive Immunology Research Center, Shahid Sadoughi University of Medical Sciences, Yazd, Iran.
E-mail: mina_poorrajab@yahoo.com

https://dx.doi.org/10.17179/excli2023-6132

This is an Open Access article distributed under the terms of the Creative Commons Attribution License (http://creativecommons.org/licenses/by/4.0/).

EXCLI Journal 2023;22:502-515 – ISSN 1611-2156
Received: April 25, 2023, accepted: June 06, 2023, published: June 14, 2023

Raw data concerning **Table 1** in the supplementary information

Supplementary Table 1: The anthropometric statistics of the type 2 diabetes study population

BMI-AST	***BMI-PLC***	***Age***	***Sex***
21.8	32.6	56	m
26.2	26.8	53	m
27.1	24	36	f
30.4	26.2	52	f
24	24.5	49	m
31.9	22.7	51	m
31.4	36.3	55	f
23.7	30.8	61	f
34.8	30	48	f
35.8	32.8	52	f
33.7	26.4	57	f
38.7	29	48	m
27.4	26.1	63	m
32.4	25.7	66	m
23.5	31.2	44	m
31	27.1	54	m
29.6	28.4	50	m
28.5	22.6	44	m
34.7	28.6	46	f
32.8	30.7	49	m
27.3	27.9	39	f
27.7	25.4	41	f
27.5	21.49	47	m
29.6	32.9	55	m
22.8	27.1	47	m
26.9	25	63	m
27.5	27.4	41	f
29	24.8	59	f
24.4	22.4	55	f
31.9	34.5	58	m
31.1	31.2	49	f
24	30	60	m
34.1	33.1	38	f
37.1	28	56	m
32.8	28.7	45	f
39.5	25.7	53	f
27.4	25.3	63	f
32.4	30.9	57	m
23.5	26.2	48	m
30	28	43	f
29.2	22.3	59	f
28.9	29.3	54	f
35.6	31	51	f
32	27.5	42	f
27	25.4	61	f
27.4	21.2	58	f
26.2	27.6	54	m

Values are means ± SD of patients. *P < 0.05 for within- and between-group change from baseline to 12 weeks. Paired t-tests were used to compare values before and after treatment in each group, whereas differences between the two groups were analyzed by one-way ANOVA. Any P-value < 0.05 was considered statistically significant. PLC: Placebo group, AST: Astaxanthin group

EXCLI Journal 2023;22:502-515 – ISSN 1611-2156
Received: April 25, 2023, accepted: June 06, 2023, published: June 14, 2023

Raw data concerning **Figure 2** in the main article

Table 2: Analysis of the relative gene expression of SIRT1 in the placebo and astaxanthin groups, before and after the intervention, compared to the control gene GAPDH

2^-dct-SIRT1-AST	***2^-dct-SIRT1-PLC***
0.0347	0.0011
0.0029	0.0448
0.0069	0.2517
0.0067	0.2349
0.0284	0.0361
0.0040	0.0328
0.0066	0.0367
0.0407	0.0608
0.0086	0.0065
0.0026	0.0166
0.0068	0.0192
0.0134	0.0179
0.1096	0.0056
0.0786	0.0064
0.0738	0.0065
0.1416	0.0065
0.3842	0.0097
0.2017	0.0136
0.4730	0.4897
0.0007	0.0005
0.0791	0.0114
0.0797	0.0529
0.0802	0.0018
0.0808	0.0340
0.0174	0.0045
0.0125	0.0055
0.0307	0.0088
0.0027	0.0267
0.0080	0.1417
0.0901	0.0156
0.3952	0.0126
0.0713	0.1517

The differences in mean values between the treatment groups were statistically significant; there was a difference between before and after intervention (p-value $\underset{\sim}{}$ 0.0022). The differences in mean values between the placebo groups were insufficient (p-value $\underset{\sim}{}$ 0.660). PLC: Placebo group, AST: Astaxanthin group

EXCLI Journal 2023;22:502-515 – ISSN 1611-2156
Received: April 25, 2023, accepted: June 06, 2023, published: June 14, 2023

Raw data concerning **Figure 3 in the main article**

Table 3: Investigation of AMPK activity in AST and placebo groups before and after supplementation

AMPK-AST	***AMPK-PLC***
12	16.2
1	10.1
4.8	3.9
4.1	3.4
17.4	11.6
16.8	7.3
4	14.7
4.3	7.3
13.6	15.2
2.4	10.1
4.5	5.6
16.3	13
5.9	11.1
6.2	12.9
18.3	14.2
11.2	14.7
6.7	12.7
4.3	4.2
8.5	8.3
7.4	4.4
1.3	9
5.7	9.6
9	7
3.6	10.3
10.2	7.5
8.5	3.7
7.3	8.3
18.7	9.9
15.3	8
16.3	12.7
5.7	17.8
16.7	18.7
13	14.5
16.6	8.6
7.7	9.5
19.1	13.5
13.9	7.3
8.1	8.4
11.3	7.6
9.8	14.6
5.8	6.5
4.6	11.2
7.6	9.8
9.9	7.5
13.8	12.4
15.2	5.1

The differences in mean values between the treatment groups were statistically significant; there was a difference between before and after intervention (p-value ~ 0.02). The differences in mean values between the placebo groups were insufficient (p-value ~ 0.82). PLC: Placebo group, AST: Astaxanthin group

EXCLI Journal 2023;22:502-515 – ISSN 1611-2156
Received: April 25, 2023, accepted: June 06, 2023, published: June 14, 2023

Raw data concerning **Tables 2** and **3 in the main article**

Table 4: Profiles of fatty acids in the serum of patients with type 2 diabetes

Patients's Name:1	***Fatty Acid***	***C10***	***C14***	***C15***	***C16***	***C16:1***	***C17***	***C18***	***C18: 1n9cis***	***C18: 1n7cis***	***C18: 2n6Cis***	***C18: 3n6***	***C18: 3n3***	***C20: 2n6***	***C20: 3n6***	***C20: 4n6***	***EPA+C22***	***IS (mg/ml sam-ple)***	***Total FA***
	Peak Area	159295	13855	6917	355562	31551	3098790	109335	226372	21582	454001	7702	27413		22104	112127	12728	6.6	
	mg/ml sample	0.34	0.03	0.01	0.75	0.07		0.23	0.48	0.05	0.96	0.02	0.06	0.00	0.05	0.24	0.03		3.3
	% of total FAs (w/w)	10.21	0.89	0.44	22.78	2.02		7.01	14.51	1.38	29.09	0.49	1.76	0.00	1.42	7.19	0.82		100.0
Patients's Name:2																			
	Peak Area	43408	6493		213475	15935	2389213	73845	123035	12401	252054				19884	102432	9419	6.6	
	mg/ml sample	0.12	0.02	0.00	0.59	0.04		0.20	0.34	0.03	0.69	0.00	0.00	0.00	0.05	0.28	0.03		2.4
	% of total FAs (w/w)	4.98	0.74	0.00	24.47	1.83		8.46	14.10	1.42	28.89	0.00	0.00	0.00	2.28	11.74	1.08		100.0
Patients's Name:3																			
	Peak Area	7598	7380		213547		2253763	79751	155470	16160	320515			9299		86843	11374	6.6	
	mg/ml sample	0.02	0.02	0.00	0.62	0.00		0.23	0.45	0.05	0.93	0.00	0.00	0.03	0.00	0.25	0.03		2.6
	% of total FAs (w/w)	0.84	0.81	0.00	23.52	0.00		8.78	17.12	1.78	35.30	0.00	0.00	1.02	0.00	9.56	1.25		100.0
Patients's Name:4																			
	Peak Area	7498	10677		222134		2177521	82337	169906	15488	242505	10990		5921	20833	103246	18801	6.6	
	mg/ml sample	0.02	0.03	0.00	0.67	0.00		0.25	0.51	0.05	0.73	0.03	0.00	0.02	0.06	0.31	0.06		2.7
	% of total FAs (w/w)	0.82	1.17	0.00	24.40	0.00		9.04	18.66	1.70	26.64	1.21	0.00	0.65	2.29	11.34	2.07		100.0
Patients's Name:5																			
	Peak Area	27876	9144		197958	18201	1619476	60759	149378	13444	292950	5745		5481	12505	65454	5506	6.6	
	mg/ml sample	0.11	0.04	0.00	0.80	0.07		0.25	0.61	0.05	1.19	0.02	0.00	0.02	0.05	0.27	0.02		3.5
	% of total FAs (w/w)	3.22	1.06	0.00	22.90	2.11		7.03	17.28	1.56	33.89	0.66	0.00	0.63	1.45	7.57	0.64		100.0
Patients's Name:6																			
	Peak Area	43610	13673	7889	342155	32901	4335678	131288	244056	17918	403719	18407	29098	11405	30619	137125	15560	6.6	
	mg/ml sample	0.07	0.02	0.01	0.52	0.05		0.20	0.37	0.03	0.61	0.03	0.04	0.02	0.05	0.21	0.02		2.2
	% of total FAs (w/w)	2.95	0.92	0.53	23.13	2.22		8.87	16.50	1.21	27.29	1.24	1.97	0.77	2.07	9.27	1.05		100.0
Patients's Name:7																			
	Peak Area	47714	14321	6462	408116	38577	3602407	135478	270548	21932	494353	17178	13315	14173	32199	175229	16690	6.6	
	mg/ml sample	0.09	0.03	0.01	0.74	0.07		0.25	0.49	0.04	0.90	0.03	0.02	0.03	0.06	0.32	0.03		3.1
	% of total FAs (w/w)	2.80	0.84	0.38	23.92	2.26		7.94	15.86	1.29	28.97	1.01	0.78	0.83	1.89	10.27	0.98		100.0
Patients's Name:8																			
	Peak Area	52891	18008	9103	448321	51580	4564095	159406	378959	35299	428931	14456	8345	13329	30450	192238	10800	6.6	
	mg/ml sample	0.08	0.03	0.01	0.65	0.07		0.23	0.55	0.05	0.62	0.02	0.01	0.02	0.04	0.28	0.02		2.7
	% of total FAs (w/w)	2.86	0.97	0.49	24.21	2.78		8.61	20.46	1.91	23.16	0.78	0.45	0.72	1.64	10.38	0.58		100.0
Patients's Name:9																			
	Peak Area	27927	9245		177373	16194	1704332	66486	139143	10575	165240				13526	65971	12205	6.6	
	mg/ml sample	0.11	0.04	0.00	0.68	0.06		0.26	0.54	0.04	0.64	0.00	0.00	0.00	0.05	0.25	0.05		2.7
	% of total FAs (w/w)	3.97	1.31	0.00	25.20	2.30		9.45	19.77	1.50	23.48	0.00	0.00	0.00	1.92	9.37	1.73		100.0
Patients's Name:10																			
	Peak Area	25704	15434	5660	343348	15241	2829931	236304	157934	12061	326651	5194			15439	103825	7720	6.6	
	mg/ml sample	0.06	0.04	0.01	0.80	0.04		0.55	0.37	0.03	0.76	0.01	0.00	0.00	0.04	0.24	0.02		2.9
	% of total FAs (w/w)	2.02	1.21	0.45	27.02	1.20		18.60	12.43	0.95	25.71	0.41	0.00	0.00	1.22	8.17	0.61		100.0

Received: April 25, 2023, accepted: June 06, 2023, published: June 14, 2023

Patients's Name:11	***Fatty Acid***	***C10***	***C14***	***C15***	***C16***	***C16:1***	***C17***	***C18***	***C18: 1n9cis***	***C18: 1n7cis***	***C18: 2n6Cis***	***C18: 3n6***	***C18: 3n3***	***C20: 2n6***	***C20: 3n6***	***C20: 4n6***	***EPA+C 22***	***IS (mg/ml sam-ple)***	***Total FA***
	Peak Area	54716	9203	5670	278311	17252	2692824	117057	263621	16902	426997	9661	12771		20399	105465	11582	6.6	
	mg/ml sample	0.13	0.02	0.01	0.68	0.04		0.29	0.64	0.04	1.04	0.02	0.03	0.00	0.05	0.26	0.03		3.3
	% of total FAs (w/w)	4.05	0.68	0.42	20.62	1.28		8.67	19.53	1.25	31.64	0.72	0.95	0.00	1.51	7.81	0.86		100.0
Patients's Name:12																			
	Peak Area	21211	12110		268376	28083	1888755	89892	165657	13967	282081	6706			22663	87142	6545	6.6	
	mg/ml sample	0.07	0.04	0.00	0.93	0.10		0.31	0.58	0.05	0.98	0.02	0.00	0.00	0.08	0.30	0.02		3.5
	% of total FAs (w/w)	2.11	1.21	0.00	26.72	2.80		8.95	16.49	1.39	28.08	0.67	0.00	0.00	2.26	8.68	0.65		100.0
Patients's Name:13																			
	Peak Area	97530	8464		214695	16673	1958585	71639	151924	9155		7761	57221			64659	15290	6.6	
	mg/ml sample	0.33	0.03	0.00	0.72	0.06		0.24	0.51	0.03	0.00	0.03	0.19	0.00	0.00	0.22	0.05		2.4
	% of total FAs (w/w)	13.64	1.18	0.00	30.03	2.33		10.02	21.25	1.28	0.00	1.09	8.00	0.00	0.00	9.04	2.14		100.0
Patients's Name:14																			
	Peak Area	12357	20177	12931	467478	31768	5832971	190770	359743	26677	663077	12329	9350	9694	31671	228427	10063	6.6	
	mg/ml sample	0.01	0.02	0.01	0.53	0.04		0.21	0.40	0.03	0.75	0.01	0.01	0.01	0.04	0.26	0.01		2.3
	% of total FAs (w/w)	0.59	0.97	0.62	22.40	1.52		9.14	17.24	1.28	31.78	0.59	0.45	0.46	1.52	10.95	0.48		100.0
Patients's Name:15																			
	Peak Area		5856		175392	11633	1880587	69232	176725	13846	288101		9694			80657	19100	6.6	
	mg/ml sample	0.00	0.02	0.00	0.61	0.04		0.24	0.62	0.05	1.01	0.00	0.03	0.00	0.00	0.28	0.07		3.0
	% of total FAs (w/w)	0.00	0.69	0.00	20.63	1.37		8.14	20.79	1.63	33.88	0.00	1.14	0.00	0.00	9.49	2.25		100.0
Patients's Name:16	***Fatty Acid***																		
	Peak Area	123473	25766	6702	492852	47558	2311750	131369	310544	21827	405884	12225	5582	6366	37567	142558	8690	6.6	
	mg/ml sample	0.35	0.07	0.02	1.40	0.14		0.37	0.88	0.06	1.15	0.03	0.02	0.02	0.11	0.40	0.02		5.1
	% of total FAs (w/w)	6.94	1.45	0.38	27.70	2.67		7.38	17.46	1.23	22.82	0.69	0.31	0.36	2.11	8.01	0.49		100.0

C10 (Decanoic acid), C14:0 (Myristic acid), C15 (Pentadecanoic acid), C16:0 (Palmitic acid), C16:1 (Palmitoleic acid), C17 (Heptadecanoic acid), C18:0 (Stearic acid), C18:1n9cis (Oleic acid), C18:1n7cis (Vaccenic acid), C18:2n6cis (Linoleic acid), C18:3n6 (γ-Linolenic acid), C18:3n3 (Alphalinolenic acid), C20:2n6 (Eicosadienoic acid), C20:3n6 (Dihomo-gamma-linolenic acid), C20:4n6 (Arachidonic acid), C20:5n3 (Eicosapentaenoic acid). LA: linoleic acid, GLA: gamma-linolenic, DGLA: dihomo-gamma-linolenic acid, AA: arachidonic acid, ALA: α-linolenic acid, EPA: eicosapentaenoic acid, FA: fatty acid
